# A single harmonised pharmacy process to improve clinical trial set-up times

**DOI:** 10.1136/ejhpharm-2024-004215

**Published:** 2024-06-27

**Authors:** Miriam Lettieri, Sophia Boydell, Andreea Chivu, Sarah Fallon, Andrew Ustianowski, Monika Cien, Claire Cole, Sophia Burgess, Carolyn Davies, Claire Keatley, Anne-Marie Peers, Maxine Syme, Deborah Sutton, Nicola Hermitage, Lydia Sutherland, Michelle Beecroft, Ali Aghabeigi, Beatriz Duran Jimenez

**Affiliations:** 1 Pharmacy, Manchester University NHS Foundation Trust, Manchester, UK; 2 Research and Innovation, Manchester University NHS Foundation Trust, Manchester, UK; 3 Pharmacy Department, The Christie NHS Foundation Trust, Manchester, Manchester, UK; 4 NIHR Clinical Research Network: Greater Manchester, Manchester University NHS Foundation Trust, Manchester, UK; 5 Pharmacy, Northern Care Alliance NHS Foundation Trust, Salford, Manchester, UK; 6 Greater Manchester Mental Health NHS Foundation Trust, Manchester, UK; 7 Pharmacy, East Lancashire Hospitals NHS Trust, Blackburn, Lancashire, UK; 8 Pharmacy, Stockport NHS Foundation Trust, Stockport, UK; 9 Tameside and Glossop Integrated Care NHS Foundation Trust, Ashton-under-Lyne, UK; 10 Pharmacy, Wrightington Wigan and Leigh NHS Foundation Trust, Wigan, Wigan, UK

**Keywords:** Clinical Trial, PHARMACY SERVICE, HOSPITAL, Practice Guideline, Quality of Health Care, Workforce

## Abstract

The UK has fallen from fourth to 10th place in the global ranking for clinical trial activities in the past 6 years. Due to the limited capacity of the clinical trial pharmacy workforce and delays in providing pharmacy approvals, pharmacy has been identified as one of the constraining services that delays the set-up and delivery of clinical trials. To tackle this problem, we developed a single pharmacy review process for multicentre trials across Greater Manchester (GM) and tested its feasibility and implementation in our region. A survey completed by each GM Trust suggests that this harmonised pharmacy review process for multicentre studies would expedite trial set-up time at each pharmacy site and standardise the pharmacy review process in GM. We therefore believe that this harmonised review process could potentially reduce pharmacy set-up time and reposition the UK in the global market for clinical trials.

## Introduction

 The Lord O’Shaughnessy review, a government commissioned review of commercial clinical trials in the UK, investigated the challenges faced by the NHS to deliver commercial clinical trials in recent years.^1^ A government response to this review was released on 22 November 2023.^2^ Recommendations included addressing the time-consuming and duplicative bureaucracy and to potentially adopt a national, centralised approach for processes, in line with the recent initiative for costing requirements. Pharmacy services were highlighted in the report as one of the supporting services constraining the delivery of clinical trials within the NHS.

The UK has seen a decline in commercial clinical trial activity, with a 44% drop in patient enrolments in the National Institute for Health & Care Research (NIHR) supported studies between 2017–18 and 2021–22. Additionally, the UK’s initiation of phase 3 trials has fallen, causing its global ranking to drop from fourth to 10th place over the same period. There are recent signs of improvement, but not to pre-pandemic levels.^3^

The NIHR Greater Manchester (GM) Clinical Research Network (CRN) was showcased in the report as an exception to this decline in delivering commercial clinical trials, with a 19% increase in study recruitment over the last 6 years compared with a 36% decrease nationally. The reasons are multifactorial; however, addressing bottlenecks in pharmacy is an objective. To improve pharmacy capacity, the NIHR GM CRN has been working closely with local sites to develop a single pharmacy regional review process for multicentre trials.

Currently, each NHS hospital site completes a separate clinical trial pharmacy risk assessment for any new clinical trial in accordance with Good Clinical Practice (GCP). This aims to identify and mitigate potential risks related to investigational medicinal product (IMP) management. The process for undertaking this assessment currently differs between sites and can be labour intensive, time consuming and leads to unnecessary duplication of work.

Introducing a single pharmacy regional review for multicentre trials would standardise the pharmacy review process, potentially reducing the time taken for pharmacy set-up. In line with the NIHR GM CRN objective to harmonise the approval processes, this work aimed to explore the feasibility and implementation of a single pharmacy review for clinical trials in GM.

## Methods and results

### Feasibility of introducing a harmonised risk assessment

First, we investigated the feasibility of introducing a GM-wide pharmacy set-up process.

A community of practice was established in the region to share ideas and exchange knowledge and challenges. This project involved 11 NHS Trusts, spanning 32 hospitals, with district to tertiary services represented.

Clinical trial pharmacy risk assessments and checklists were requested from each Trust/hospital. Only three large Trusts had established paperwork which were provided. Checklists and risk assessments varied considerably across Trusts. Due to the variety of services offered across Trusts in terms of clinical delivery (district vs tertiary centres), research facilities available (in situ vs partnerships), trial types (including early phase, Genetically Modified Organisms (GMO) and Advanced Therapy Investigational Medicinal Products (ATIMPs)) and medical specialities, we were confident that all pharmacy specialist services were captured.

### Development of a harmonised risk assessment process

The information obtained from the previous phase of the study was used to develop a comprehensive pharmacy risk assessment which reflected the need of each specialist area and/or patient population. This assessment and associated review process was further refined following feedback received from the GM community of practice.

The resulting harmonised risk assessment is shown in the online supplemental material S1. It encompasses 13 different sections, each focussing on a specific aspect of the review including IMP details, drug preparation requirements, dispensing and handling, funding and blinding procedures. The delivery of ATIMP clinical trials and the use of home delivery were also included. The GM harmonised risk assessment has been designed to facilitate the assessment of multicentre trials; however, it still meets all the requirements to be used for single-centre trials and it is also used for this purpose.

The GM pharmacy review process involves a single pharmacist/senior technician in the region completing most of the risk assessment (online supplemental tab 1 to 12) by reviewing the protocol, Investigator Brochure (IB), Patient Information Sheet (PIS), pharmacy manual and other relevant documentation. The reviewer also liaises with sponsors/Contract Research Organisations (CROs) to clarify and mitigate identified risks and compiles a detailed pharmacy overview for the trial to be used at local sites. Pharmacy clinical trial reviewers are specialist clinical trial pharmacy staff. In the UK, their role and responsibilities which are delegated by the principal investigator for each study are defined in the Professional Guidance on Pharmacy Services for Clinical Trials provided by the Royal Pharmaceutical Society.^4^

Each risk and solution is listed in the 'summary of risks' section, allowing each site to review and address these according to local practice. The completed risk assessment is made available to all other participating sites who would only need to complete the final section of the assessment to address any site-specific issues (‘Site Specific Details’ - online supplemental tab 13).

The sponsor will be informed of this collaborative working arrangement between selected hospitals before work commences, and risk assessment and/or any other study information documents would only be shared across hospitals after a confidential disclosure agreement has been put in place.

**Figure 1:**
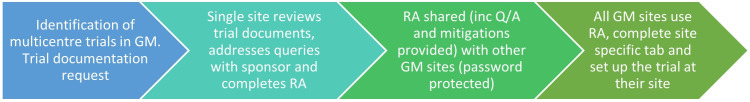
Single pharmacy review process for multicentre trials. GM, Greater Manchester; RA, risk assessment; Q/A, questions/answers.

A flow diagram that summarises this process is shown in figure 1. Further details on how to complete this harmonised pharmacy review is described in the online supplemental material S2.

### Implementation of a GM pharmacy review process

A survey was designed to identify possible benefits and challenges in the implementation of this standardised process.

Ten pharmacy professionals representing 9 of the 11 Trusts in GM contributed to the GM community of practice and seven took part in this survey (at least one representative per Trust). All agreed that a harmonised pharmacy review process for multicentre studies would expedite trial set-up time at their site and standardise the pharmacy review process in GM. All were willing to implement the process at their hospital. In addition, the majority (6 of 7) believed it would be useful to review trial amendments using this harmonised process. Interestingly, 3 of 7 representatives (43%) did not agree that this could increase clinical trial pharmacy capacity at their hospital and reasons for this view should be further investigated.

It was concluded that this process would most likely shorten the time spent by pharmacy to address queries with sponsors (six voted very likely and one voted likely) and speed up the initial pharmacy feasibility assessment (initial review of trial documents and pharmacy manual) (four voted very likely and three voted somewhat likely). It is unsurprising that there were no prominent views on whether the process would speed up writing/approving pharmacy-specific documents (ie, dispensing and handling procedure, trial prescription, accountability logs) as these are site-specific activities (two very likely, three somewhat likely, two neither likely nor unlikely).

## Discussion

We have developed and are in the process of implementing a single pharmacy risk assessment for multicentre clinical trials across GM in line with the UK government’s objective to address the slow set-up times and pharmacy capacity issues highlighted nationally.^5^

Our study suggests that this comprehensive single pharmacy review process can improve clinical trial set-up times within pharmacy and avoid duplication of work involving multiple pharmacy staff across Trusts without compromising safety and GCP. Setting up trials faster would also benefit sponsors by saving time and money in return.

The potential benefits of using a single pharmacy review have been recognised for at least 10 years in the UK.^6^ This culminated in the development of the Health Research Authority (HRA) Pharmacy Assurance process in 2017. The HRA coordinated a standardised single technical pharmacy review to streamline study set-up in pharmacy departments at hosting sites. Such a development has been hugely welcomed; however, nationally this process has not yet achieved its full potential and does not currently provide the full functionality of this regional process. When undertaken, the HRA Pharmacy Assurance is an important aid to determining feasibility but currently does not replace a full harmonised risk assessment to the same degree and does not include all IMP handling tasks and study amendments. The GM pharmacy risk assessment proposed in this study aims to dovetail with the HRA Pharmacy Assurance to address these deficiencies.

### Limitations

Although this approach could facilitate trial set-up in pharmacy, we are yet to fully implement it across the region and subsequent data will be required to validate this model in the long-term across a variety of study types.

We have identified operational limitations associated with this project which are currently slowing its implementation:

1. A shared electronic platform which can be accessed by all Trusts and complies with GCP and NHS governance is required. We are currently testing the feasibility of using FutureNHS as both a repository and a working environment.
2. This strategic project has been funded and hosted by GM CRN, but a more permanent solution needs to be agreed for the process to be appropriately managed moving forward.

To conclude, the proposed harmonised clinical trial pharmacy review process has the potential to address the inefficient and repetitive pharmacy workload highlighted nationally. We are in the process of implementing this across GM but it could be implemented nationally if it is shown to be an effective tool, and perhaps integrated with the current HRA Pharmacy Assurance process for all multicentre trials. Expediting clinical trial set-up in pharmacy would reduce overall trial opening times and possibly increase NHS pharmacy capacity to deliver more studies. As a result, this will facilitate the availability of innovative treatments to patients and increase the appeal of the UK as a leader in the delivery of clinical research.

## Supplementary material

10.1136/ejhpharm-2024-004215 online supplemental file 1

10.1136/ejhpharm-2024-004215 online supplemental file 2

10.1136/ejhpharm-2024-004215 online supplemental file 3

## Data Availability

All data relevant to the study are included in the article or uploaded as supplementary information.
