# Supplementary material for: A single harmonised pharmacy process to improve clinical trial set-up times
Source: Eur J Hosp Pharm. 2024 Jun 27;33(2):e004215. doi: 10.1136/ejhpharm-2024-004215 (PMC13018745; doi:10.1136/ejhpharm-2024-004215)
Supplement: online supplemental file 1 [file ejhpharm-33-2-s001.pdf]

Guidance

The **Site specific tab** can be completed by Trusts in GM who are also opening to the study. This can include specific information as per local requirements and can replace their own process at site if they choose.

Each amendment will be reviewed in the **Amendments tab**. The changes will be implemented across the checklist and document versions updated.

Drop-down options are provided for some of the questions to ensure standardisation.

*****Before reviewing this checklist please ensure you have the most up to date documentation and that this matches the versions used in this review document*****

*****This document is version controlled. When processing ensure you have the latest version number which is found in the Initial review tab*****

Designed and built by:

Andreea Chivu - Senior Pharmacist Clinical Trials - NHS Manchester Foundation Trust
Sophia Boydell - Research and Innovation Manager - NHS Manchester Foundation Trust
Miriam Lettieri - Clinical Trials Pharmacist - NHS Manchetser Foundation Trust
Beatriz Duran - Consultant Pharmacist - Clinical Trials and ATMPs - NHS Manchester Foundation Trust

Version: **1.3 23.02.2024**

<u>1. Initial review</u> ***(to complete tabs 1 - 12 on behalf on all GM sites)***

Initial review completed by:

Name:	
Position:	
NHS Trust:	
Date completed:	
Version:	
Superseded version:	

Participating NHS Trusts in GM ***(name and contact details of other known sites in GM)***

Participating NHS Trusts in UK ***(name and contact details of other known sites in UK)***

Documents reviewed and approvals received at the time of this assessment

Protocol version and date	
Date received	
Pharmacy Manual Version and date	
Date received	
IB Version and date	
Date received	
Other documents used *(please specify i.e. PIS, SmPC, IRAS form etc.)*	
Version and date	
Other documents used *(please specify i.e. PIS, SmPC, IRAS form etc.)*	
Version and date	
MHRA Approval received	
Date received	
REC Approval received	
Date received	
HRA Approval received	
Date received	

2. Study details

General information	
Study acronym	
Full protocol title	
Protocol number	
IRAS number	
Type of funding *(commercial, non-commercial)*	Please select
Sponsor name	
CRO (if applicable)	
Clinical speciality / Indication	
Phase of Study	Please select
(If 'other', specify)	
Risk-based type (*A, B, C) - if known, please state if type A*	Please select
Set up type as per industry costing template *(A, B,C, D, E, F, G)*	Please select
Study design *(open label, blinded)*	Please select
Types and duration of study phases: screening period + treatment period + follow up	
Additional study information *(i.e. IMP vs placebo / multiple arms / sub-studies etc.)*	
Potential number of participants in the study in GM / UK	
Recruitment period	

3. Amendments

NB: When implementing a new Amendment, please update each relevant affected section and update the Initial review tab with the most current RA version number

Amendment Number:

Date received	
Amendment type	Please select
Amendment category	Please select
Amendment summary (*see IRAS form for summary of changes*)	
Documents affected (*state names and version numbers*)	
**Approvals**	
MHRA Approval received	Please select
Date received	
REC Approval received	Please select
Date received	
HRA Approval received	Please select
Date received	
Other approvals received *(state which)*	
Date received	
Date amendment processed	
Has this amendment impacted on any of the risk previously identified? *if Y, update summary of risks tab*	
State pharmacy documents which may require amending<br>*(i.e. labels, prescription, acc logs, unblinding document, financial agreement etc)*	

Amendment Number:

Date received	
Amendment type	Please select
Amendment category	Please select
Amendment summary (*see IRAS form for summary of changes*)	
Documents affected (*state names and version numbers*)	
**Approvals**	
MHRA Approval received	Please select
Date received	
REC Approval received	Please select
Date received	
HRA Approval received	Please select
Date received	
Other approvals received *(state which)*	
Date received	
Date amendment processed	
Has this amendment impacted on any of the risk previously identified? *if Y, update summary of risks tab*	
State pharmacy documents which may require amending<br>*(i.e. labels, prescription, acc logs, unblinding document, financial agreement etc)*	

Amendment Number:

Date received	
Amendment type	Please select
Amendment category	Please select

Amendment summary (*see IRAS form for summary of changes*)	
Documents affected (*state names and version numbers*)	
**Approvals**	
MHRA Approval received	Please select
Date received	
REC Approval received	Please select
Date received	
HRA Approval received	Please select
Date received	
Other approvals received *(state which)*	
Date received	
Date amendment processed	
Has this amendment impacted on any of the risk previously identified? *if Y, update summary of risks tab*	
State pharmacy documents which may require amending<br>*(i.e. labels, prescription, acc logs, unblinding document, financial agreement etc)*	

**Amendment Number:**	
Date received	
Amendment type	Please select
Amendment category	Please select
Amendment summary (*see IRAS form for summary of changes*)	
Documents affected (*state names and version numbers*)	
**Approvals**	
MHRA Approval received	Please select
Date received	
REC Approval received	Please select
Date received	
HRA Approval received	Please select
Date received	
Other approvals received *(state which)*	
Date received	
Date amendment processed	
Has this amendment impacted on any of the risk previously identified? *if Y, update summary of risks tab*	
State pharmacy documents which may require amending<br>*(i.e. labels, prescription, acc logs, unblinding document, financial agreement etc)*	

4. IMP details

Description	IMP 1	IMP X	Additional comments
Full name of product			
Strength			
Form and presentation, including total quantity per pack *(i.e. xNo cps / bottle, xml / ampoule)*			
Admin route			
Dose / dosing schedule			
Frequency			
State any dose escalation / reduction allowed			
missed dose			
Is the IMP licensed or unlicensed in the UK?	Please select	Please select	
If licensed, is it used for the same indication / age group?			
Is a Home Office Licence required?	Please select	Please select	
Add additional comments if needed (i.e. restricted use, blueTeq, etc.)			
State generic / band name included in the IRAS application			
Concomitant medications			
Supportive medication required? *(state for each IMP)*			
Permitted medications			
Prohibited medications			
Rescue medication required? *(state for each IMP)*			
Mechanism of action			
Add any clinical risk associated with the use of the IMP			

Preparation and Administration	IMP 1	IMP X	Additional comments
Does the IMP need reconstitution before administration *(if yes, further details in section 7)*	Please select	Please select	
Does the IMP need dilution before administration *(if yes, further details in section 7)*	Please select	Please select	
Does the IMP require aseptic preparation *(if yes, further details in section 7)*	Please select	Please select	
Are specific brands of ancillaries/diluents mandated by the pharmacy manual for administration *(if Y, further details in section 7)*	Please select	Please select	
Does IMP reconstitution need staff training *(Consider multiple steps/multiple staff need involvement/special handling precautions)*	Please select	Please select	
Are there any special precautions for administration *(Think syringe drivers, pt operated administration pumps, etc. and staff/subject training needs)*			
Administration duration ands rate *(hours/minutes)*			
Max treatment duration (i.e. number of cycles)			
Is dose banding permitted?	Please select	Please select	
- If yes, what process/guidance should be followed?			
- If yes, what dose capping protocols will be permitted?			
Any special requirements for administration? i.e. clinical monitoring of participant post-dose			

Storage	IMP 1	IMP X	Additional comments
Is the IMP a controlled drug? *(state schedule)*	Please select	Please select	
Storage requirements *(specify range in the comments)*			
Temperature excursions reporting procedure as requested by the sponsor			
Is storage out of Pharmacy required?	Please select	Please select	
Does transport to ward/clinic need temperature monitoring? **If yes, enter individual site arrangements in tab 13*	Please select	Please select	

Supply	IMP 1	IMP X	Additional comments

IMP supplied by?	Please select	Please select	
- If 'other' please specify………			
Packaging of product and size *e.g. Primary: in HDPE bottles with child resistant cap, Secondary: 1 carton (kit) contains 2 bottles*			
Shelf life/expiry			
Is the QP release in line with UK requirements?	Please select	Please select	
Post-trial access arrangement for product provision *(e.g.Compassionate supply, planned extension study, no provision by sponsor)*			

**Handling requirements**	**IMP 1**	**IMP X**	**Additional comments**
Are there any special handling requirements? Select Category IMP falls into:	Please select	Please select	
Is Manufacturer Safety Data Sheet (MSDS) available?	Please select	Please select	
Do staff need special training for IMP handling *(think IMP receipt, dispensing, return/destruction process)*	Please select	Please select	
Does the sponsor supply spilling kits?	Please select	Please select	

**Risk(s) identified:**

5. NIMP details

Is this tab applicable for this trial? Please select

Description	NIMP 1	NIMP X	Additional comments
Full name of product			
Strength			
Form and presentation, including total quantity per pack *(i.e. xNo cps / bottle, xml / a mpoule)*			
Admin route			
Dose / dosing schedule			
Frequency			
State any dose escalation / reduction allowed			
State generic / band name included in the IRAS application			
Product licence status (licenced/unlicenced/used off-label)			
Supportive medication required? *(state for each NIMP)*			
Rescue medication required? *(state for each NIMP)*			
Add any clinical risk associated with the use of the IMP			

Preparation and Administration	NIMP 1	NIMP X	Additional comments
Does the NIMP need reconstitution before administration? (if yes, further details in section 7)	Please select	Please select	
Does the NIMP need dilution before administration (if yes, further details in section 7)	Please select	Please select	
Does the NIMP require aseptic preparation *(if yes, further details in section 7)*	Please select	Please select	
Are specific brands of ancillaries/diluents mandated by the pharamacy manual for administration *(if Y, further details in section 7)*	Please select	Please select	
Are there any special precautions for administration *(Think syringe drivers, pt operated administration pumps, etc. and staff/subject training needs)*			
Administration duration *(hours/minutes)*			
Is dose banding permited?	Please select	Please select	
- If yes, what process/guidance should be followed?			
- If yes, what dose capping protocols will be permitted?			

Storage	NIMP 1	NIMP X	Additional comments
Is the NIMP a controlled drug? *(state schedule)*	Please select	Please select	
Storage requirements	Please select	Please select	

Supply	NIMP 1	NIMP X	Additional comments
NIMP supplied by?	Please select	Please select	
- If 'other' please specify.........			
Packaging of product and size *e.g. Primary: in HDPE bottles with child resistant cap, Secondary: 1 carton (kit) contains 2 bottles*			
Shelf life/expiry			

Handling requirements	NIMP 1	NIMP X	Additional comments
Are there any special handling requirements? Select Category NIMP falls into:	Please select	Please select	
Is Manufacturer Safety Data Sheet (MSDS) available?	Please select	Please select	
Does staff need special training for NIMP handling *(i.e. IMP receipt, dispensing, return/destruction process)*	Please select	Please select	
Does the sponsor supply spilling kits?	Please select	Please select	

Risk(s) identified:

6. ATIMP/Early phase/GMO

Is this tab applicable for this trial? Please select

Properties and contract	ATIMP 1	ATIMP X
State ATIMP name and presentation (strength, number of units, pharmaceutical form etc)		
Type of ATMP: Gene therapy (in-vivo or ex-vivo), cellular product, tissue engineered product		
Is there any device involved in the delivery of ATMP?		
Dose - has this been tested in vivo? In humans? - see IB		
Treatment schedule, has this been tested in vivo? In humans? - see IB		
Indication and action mechanism		
Nature of target (Please provide available knowledge on the nature of the target and variation between individuals)		
Is ATIMP availability guaranteed for the duration of the study? Has this been covered in the contract?		
What is the expected total exposure of the associated drug and the anticipated plasma concentrations?<br>Has this been covered in the IB, in animal studies for phase 1 first in human, and phase 1-2 for later phase studies		
Reliability of very small doses (if applicable) Is there a need for demonstration that the intended formulation of the doses to be administered provides the intended dose?		
If GMO: Biosafety Level and Containment level (complete the GMO section below)		
Are there any issues with compatibility of ATIMP?		

Product Pathway	ATIMP 1	ATIMP X
Where is the ATIMP sourced and manufactured? (any risk associated to this? Importing/exporting licence)		
Will the QP release certificate be provided with the shipment?		
Storage requirements		
Where is this product prepared? Stem cell lab, theatres, aseptic unit, etc		
Are any ancillaries mandated to use? Will they be provided by the sponsor? Does the preparation method need to be validated?		
What is the shelf life of the ATIMPs? When received and after preparation any specific store requirements after preparation?		
Once prepared for administration, how much time is there to administer ATIMP to the subject?		
Is there a specific spill kit mandated to use by the sponsor? Is the sponsor providing it?		
What is the route of administration?		
What is the rate of administration?		
What is the estimation of first dose?		
What method is used to calculate the dose?		
Who will be responsible for checking the calculations at dosing times (if applicable)?		
What is the dose escalation strategy (if applicable)?		
Who will administer the first dose? (eg: PI / researcher)		
Which members of the trial team will be present at the administration of first dose?<br>(can be added to Site specific tab if not defined by Protocol)		
Is there any monitoring required during and after administration? Is there a period of observation after administration?		
Is the sponsor providing supportive drugs/concomitant medication?		
For Early Phase: Describe the strategy to manage likely adverse events or adverse reactions (see Site specific tab for contingency plans)		
Will an antidote be immediately available on site? (If Yes, please provide details of the antidote and the plan for subject management;<br>If No, please justify and detail contingency plan)		
Is there any specific handling requirement? For GMO see section below		
Is there any specific waste management requirement? For GMO see section below		
Is there any specific risk to the staff handling the product?		

Where will this product be store on site? Is it safe and secure? Will it need to be transported to storage site?<br>Any specific requirements for transportation to the storage location?		
Local assessment: how will the storage be monitored? Who checks this prior administration? Is there a checklist?		
Does it need Aseptics processing for reconstitution/dilution/administration (if Yes see Aseptic requirements tab)		

**Criteria for stopping the trial**	**ATIMP 1**	**ATIMP X**
The criteria and protocol for premature discontinuation of ATIMP in an individual. This should include clear details on clinical parameters		

**GMO**	**IMP/ATIMP 1**	**IMP/ATIMP X**
How long will the GM organism persist following administration?		
What is the route and level of shedding?		
How will shedding be monitored and contained?		
What are the consequences for other body systems (non-target) from the systemic administration of the gene product?		
What is the normal mode of transmission of the GMO? Can transmission happen through other routes such as needlestick injuries?<br>If so, how would these be minimised?		
What are the possible consequences of an accidental exposure? (To the person administering the gene therapy product)		
What samples are required to be taken from the patients following administration of the gene therapy product?		
(Specify where the samples will be taken for analysis, how they will be transported and who will analyse them)		
What Personal Protective Equipment will they be required at each stage?		
(Specify the PPE required during sampling, ATMP administration and caring for the patient post administration)		
What decontamination or disposal arrangements are in place for PPE?		
Does the nature of this work preclude it being undertaken by any workers who have a serious skin condition (e.g. eczema) or other<br>health problems that might make them more susceptible to infection?		
Specify any health surveillance requirements for staff involved in the work		
Will visitors to patient be allowed? How will their risk of exposure be minimised? What information will they be provided with		
Other than standard arrangements, are any additional safety measures or procedures required for cleaning the patient's room,<br>bed linen or laundry or disposing of dressings/nappies etc?		
Describe whether there are specific procedures to be followed in the event of the patient requiring resuscitation		
Are there specific actions to be taken in the event of death of the patient before the end of the treatment period?		
Waste and Spillage		
What are the stages at which contaminated waste is generated and what types/quantities of items will be produced?		
How would residual/unused GMO be disposed?		
How will GMO waste be contained during transport and prior to disposal?		
Will GMO waste be inactivated and how?		
Will there be any waste still subject to GMO legislation relating to transport and disposal?		
Describe the procedures in place for dealing with spillages of the product		
Describe the procedures in place for an accidental exposure eg. eye splash, percutaneous inoculation.<br>(immediate action, when and to whom to report the incident, medical intervention or prophylaxis)		

**Risk(s) identified:**

7. Injectable preparation requirements

Is this tab applicable for this trial? **Please select**

Does preparation of the IMP/non-IMP used in the study require an aseptic service unit (ASU)?	Please select
Could the IMP/non-IMP used in the study be prepared on the ward using aseptic technique?	Please select

If you answered yes to any of the above questions please complete the following tables:

**IMP/NIMP details**	**IMP 1**	**IMP X**	**NIMP 1**	**NIMP X**
Prepared by ASU or ward	Please select	Please select	Please select	Please select
NPSA Risk Assessment Score	Please select	Please select	Please select	Please select
Pharmaceutical form				
Drug type	Please select	Please select	Please select	Please select
Drug storage requirements	Please select	Please select	Please select	Please select

**Reconstitution details (as per trial protocol)**	**IMP 1**	**IMP X**	**NIMP 1**	**NIMP X**
Method				
Reconstitution fluid				
Concentration range				
Expiry & storage after reconstitution				
Any other sponsor requirements *(i.e. keep used vials)*				

**Dose Preparation Method (as per trial protocol)**	**IMP 1**	**IMP X**	**NIMP 1**	**NIMP X**
Method				
Final diluent and volume				
Additional requirements for preparation *(e.g. specific consumables required)*				
Expiry & storage after preparation				
Additional products required (e.g. Flushes, Non IMP drugs)				

**Occupational exposure controls**	**IMP 1**	**IMP X**	**NIMP 1**	**NIMP X**
Process in case of eye contact				
Process in case of Skin contact				
Process in case of Inhalation				
Process in case of Spillage				
Number of doses and frequency required for each patient				

**Ancillaries for drug preparation & administration**	**Ancillary 1**	**Ancillary X**
Brand / ref code		
Supplied by		
Available in the UK as NHS supply?		
Any compatibility considerations *(e.g. needles, latex, bungs etc.)*		
Any specific storage / other requirements		

**Risk(s) identified:**

8. Excess Treatment Costs

NIMP/ other medications

	Yes/No/N/A	if Y, specifiy Name/strength/form/quantity
Does site need to cover any NIMP cost?	Please select	
Does site need to cover any supportive medication cost?	Please select	
Does site need to cover any rescue medication cost?	Please select	

Additional time & costs considerations

	Yes/No/N/A	Add comments
Does NIMP need accountability / special requirements?	Please select	
Is the IMP an high cost medication?	Please select	
Does NHS England need to approve it?	Please select	
Is it standard of care?	Please select	
Do site need to cover ancillaries costs	Please select	
Is any Aseptic Unit validation required?	Please select	
Are there any reinbursements by the sponsor?	Please select	
Does dispensing of IMP require out of hours?	Please select	
Does pharamcy staff need to complete any study specific training? *(i.e. IXRS/IVRS)*	Please select	
Postage/courrier for IMP delivery to patients?	Please select	
End of trial arrangement *(i.e. post trial access to IMP: compassionate supply, planned extension study, no provision by sponsor etc.)*		

Risk(s) identified:

9. Pharmacy requirements

IMP Dispensing	IMP 1	IMP X	Additional comments
IMP name, strength and formulation			
In which setting is the IMP administered?	Please select	Please select	
Does the IMP require dispensing out of hours?	Please select	Please select	
Number of dispensing visits per participant & frequency			
Number of kits / packs per dispensing			
Does dispensing involve packing-down?	Please select	Please select	
If IMP comes in vials and multiple vials are packed in a carton, is dispensing per vial or carton?			
Is IMP label compliant with Annex 13?	Please select	Please select	
Is a pharmacy dispensing label required?	Please select	Please select	
Will IMP require re-labelling at any point? *(i.e. new expiry date, blinding)*	Please select	Please select	
Drug-food interactions (if so, are they mentioned in the PIS?)	Please select	Please select	
If there are drug-food interactions, do these need to be added to the IMP label? *(protocol/PIS)*	Please select	Please select	
Are there any cautions/warnings/admin instructions required on the labels (i.e. BNF cautions)?			
Is there any written material to be given to particpant with treatment i.e contact/unblinding card, diary, instructions etc.)?			
Pre-Conditioning treatment required to be dispensed by pharmacy ? *(if applicable)*	Please select	Please select	
Are ancillaries required to be supplied by pharmacy? *(if Y, specify types and numbers)*	Please select	Please select	

Treatment allocation / randomisation	IMP 1	IMP X	Additional comments
Process for treatment allocation / randomisation *(IVRS, manual allocation, who does the randomisation - RN/Pharmacy etc? are screening and randomisation numbers different?)*			
Will Pharmacy be involved in treatment allocation/randomisation?	Please select	Please select	
How will Pharmacy be notified of treatment allocation? (IVRS, email alert, etc)			
Can randomisation / kit allocation be done in advance of patient visit?	Please select	Please select	
Does pharamcy staff need to complete online training to get IWRS access?	Please select	Please select	
Is kit allocation controlled via an IWRS?	Please select	Please select	

Pharmacy Clinical checks	IMP 1	IMP X	Additional comments
Does dispensing require a pharmacist check / drug release? *(i.e. bloods, drug interactions, treatment allocation)*	Please select	Please select	
Does pharmacy need to calculate dose?	Please select	Please select	
- If yes, detail method (i.e. BSA formula, dose table provided etc.)			
What dose capping/rounding are permitted?			
Are different formulations available if required *(i.e. paediatric population, inability to swallow)*			

Accountabilities	IMP 1	IMP X	Additional comments
Does the sponsor supply their own logs? *(If so, which ones?)*	Please select	Please select	
Are sites allowed to use their own accountability logs?	Please select	Please select	
Is a release form/prescription supplied by sponsor?	Please select	Please select	
Are sites allowed to use their own prescriptions?	Please select	Please select	
Other type of accountability logs required and will sponsor supply them?	Please select	Please select	
Are any compliance calculations / returns counting required by sponsor?	Please select	Please select	

Supply / Shipments	IMP 1	IMP X	Additional comments
When will there be the first delivery? *(e.g. at patient screening, prior to screening, how far in advance etc)*			
What is the ordering & re-ordering process *(IVRS, CRA request in advance, etc)*			

Shipment acknowledgement *(how is this done: IVRS, email, etc)*			
Is there homecare involvement? *(if Y, please complete homecare tab)*	Please select	Please select	
Will shipment boxes need to be returned to the sponsor? *(i.e. credo boxes)*	Please select	Please select	
Is temperature monitoring device supplied with shipment?	Please select	Please select	
Does IMP require to be courrier to patient? If so, is temperature monitoring required?	Please select	Please select	
What is the lead time for delivery of IMP once order is generated?			

**Returns and Disposal**	**IMP 1**	**IMP X**	**Additional comments**
Disposal requirement as specified by sponsor (returns/destruction)			
What will need to be retained in pharmacy until sponsor reconciliation *(used/damaged IMP, IMP packaging?)*			
What is the batch recall procedure?			

**Risk(s) identified:**

10. Homecare

**Is this tab applicable for this trial?**	**Please select**

**Documentation review**	
Type of Homecare identified	Please select
Which IMP(s)/NIMP(s) will be included in Homecare?	
Is the option of Homecare visits included in the Protocol and the Participant Information Sheet (PIS)?	Please select
iCT includes all applicable homecare costs?	
State any issues identified when reviewing the sponsor/homecare documents (i.e. chain of custody form, dosing/calculation form etc.)	
Will there be a SLA with each site involved?	
Is there a comunication plan (how is the PI infomred about AEs and IMP management issues, i.e. dosing incidents?)	

**Operational considerations**	
Please state any pre-meds/Anaphylaxis/rescue meds required	
(If applicable, provide details on how supply will be managed)	
Please state any consumables required (e.g., diluents, filters etc.)	
(If applicable, provide details on how supply will be managed)	
Please state any special consideration for transit (e.g., limited time, stability, light, temperature, packaging)	
What is process for booking transport? (e.g., courier and who is responsible for this)	
Who will complete and receive booking forms?	
Special considerations for safe handling in patient’s home?	
Are there any blinding considerations?	
Storage of IMPs or NIMPs required at patient’s home? (If yes, provide details on how secure storage will be maintained)	
Temperature monitoring required for transit or home? (If yes, please give details)	
Does Homecare provider have a system for downloading reports e.g., Temptales and relaying these to site? (If yes, please give details)	
Does Homecare provider have a process to contact sponsor DIRECTLY in the event of a temperature excursion during transit/at home? (please give details)	
Is a Spillage kit required? Provide details.	
What are the disposal arrangements for IMPs/ NIMPs? If return to site is required, provide details of process.	
BH arrangements (no pharmacy provision)	
Pharmacy trials/aseptics provision required for on site visits If yes, please provide details	
Do the forms and processes provide clear evidence of sponsor and PI oversight of the homecare provision and clear communication channels to both for resolution of queries?	

**Risk(s) identified:**

11. Blinding/Unblinding

Is this tab applicable for this trial? **Please select**

Who is blinded to the study?	
Is a site-blinding plan required?	
What system is in place for unblinding? *(i.e. IWRS, envelope)*	
Will Pharmacy be involved in the process of unblinding?	Please select
Detail process for unblinding *(during working hours, out of hours, in an emergency)*	
Sponsor 24h contact details	
If local pharmacies will be involved in re-packing and/or re-labelling medication to blind, please give details	

**Risk(s) identified:**

12. Summary of Risks

	Risk(s) identified	Actions taken to minimise risks
**Section 4: IMP details**	0	0
**Section 5: NIMP details**	0	0
**Section 6: ATIMP & phase 1**	0	0
**Section 7: Injectable preparation**	0	0
**Section 8: Excess Treatment costs**	0	0
**Section 9: Pharmacy requirements**	0	0
**Section 10: Homecare**	0	0
**Section 11: Blinding-Unblinding**	0	0

13. Site specific details - *to be completed by each site*

Before reviewing this checklist please ensure you have the most up to date documentation and that this matches the versions used to complete checklist sections 1 - 12

Does current documentation match versions used to complete checklists 1-12, including any amendments?	Please select
Site specific assessment completed by (sign and date):	

**Research team contacts**	
R&I Reference No.	
PI	
Lead Research nurse	
Study Coordinator/ Clinical Trial Manager	
Clinical pharmacist covering the specialist area (incl. Aseptics):	
Date protocol sent to specialist area	
Name and contact details of Sponsor CRA	
Name and contact details of CRO *(if applicable)*	
Name and contact details of Monitor	

**Study details**	
Anticipated study start date	
Recruitment target for site	
Anticipated end date for recruitment	
How many participants per month are expected?	
Participants to be recruited from inpatients/outpatients/both?	
Which part of the study will site be taking part in?	

**Other facilities / teams involvement**	
Where will the IMPs be dispensed?	
Does a trust committee need to be involved? (i.e. antibiotics)	
Have the nurses confirmed that they undertsand the IP administration requirements including use of specifc auxiliaries?	
Does the product require aseptic preparation in the Aseptics unit?	Please select
If Yes, give details	
Can the product be prepared/reconstituted on ward/pharmacy?	Please select
If Yes, give details	
Has an agreement been reached with sponsor regarding area of preparation?	
Is transport to another site necessary? *If so, give details.*	
What is the process for IMP returns?	
Any issue identified regarding pharmacy capacity and capability of dispensing IMPs	

**Practical considerations**	
First delivery expected	
Size of shipment	
Supply method (i.e *IMP only/ready to be administered)*	
Number of dispensing per supply *(if applicable)*	
Additional label required as per local policies? *(i.e fridge label, cytotoxic warning etc.)*	
List products which are locally sourced and how / where they are issued (if any)	
Storage location	
Storage of IMP outside pharmacy	

Are paper temperature logs required? If so, please give details i.e. sponsor/Trust to provide	
What is the temperature monitoring arrangements for transport from pharmacy? *(if applicable)*	
Is the release form/prescription required to be supplied by Pharmacy? And if so, does it need sponsor approval?	
What is the process for wet ink prescriptions to be delivered to pharmacy?	
Clinical check required?	
Multi-site / One site *(consider this for shipments/IXRS)*	
What is the sponsor's & Trusts arrangement for the safe and secure handling of the controlled drug?	
Are arrangements needed to deliver IMP to participant? (if so, please provide details)	
Do pharmacy need to monitor IMP stock levels and reorder as needed?	
Has the IMP/NIMP supply chain been established?	
Any other issues e.g. does the trial deviate from Trust policies or procedures? Is standard care arm in line with local practice? etc.	

Review and approval timelines

Actions to be taken to provide support	
If unable to approve/support trial, state reason	
Date first informed of Study	
Date Protocol received	
Official request from R&I for support/costs	
Additional information required?	
Details of additional information requested	
Date all additional information received	
Date costs provided to R&I	
Trust committee approval required?<br>If other, please specify…..	Please select
Approval date	
Risk Assessment with HTA Designated individual complete?	
Date pharmacy feasibility completed	
SIV date	
Commercial Trials: Date costing template reviewed	
Arrangements for invoicing	
Date pharmacy approval sent to R&I	
Green light issued by	
Date Pharmacy Green light email sent *(email to include PI, CRA, lead research nurse, R&I)*	

Homecare (complete if applicable)

Reason for homecare review	Please select
Type of Homecare identified	Please select
Service Level Agreement in place - IMP tasks documented and reviewed	Please select
Aseptics/directorate/management pharmacist/Homecare notified?	Please select
Applicable Risk Assessments completed e.g Direct to Patient	Please select
Please state any site-specific issues identified	

Amendments

Amendment type and date	
State pharmacy documents which required amending<br>*(i.e. labels, prescription, acc logs, unblinding document, financial agreement etc)*	
Date Trust approval/no objection given	
