# Supplementary material for: A single harmonised pharmacy process to improve clinical trial set-up times
Source: Eur J Hosp Pharm. 2024 Jun 27;33(2):e004215. doi: 10.1136/ejhpharm-2024-004215 (PMC13018745; doi:10.1136/ejhpharm-2024-004215)
Supplement: online supplemental file 2 [file ejhpharm-33-2-s002.pdf]

Guideline for Completing the Pharmacy CTIMP Risk Assessment for the set-up of Clinical Trials delivered across Greater Manchester

Background

Setting up and delivering clinical trials within the NHS involves several different departments and each of them should consider their responsibilities and duties with respect to the trial protocol and the level of risk in relation to this. The IMP management is usually delegated to pharmacy. As part of the IMP management review, pharmacy complete a clinical trial risk assessment to assess the potential hazards to the participants, the organisation and the staff associated with a trial. Once risks are identified and categorised, the risk assessment is used by pharmacy to mitigate those risks and propose solutions in line with the trial protocol, GCP, Research Governance Framework, legislations and local policies.

Purpose

The purpose of this document is to provide clear guidance for the completion of the GM clinical trials pharmacy risk assessment, to centralise and streamline the pharmacy review of multicentre clinical trials in set-up across GM. The aim is to improve pharmacy set-up time by removing repetition of work from participating sites.

Objectives

- To standardise the pharmacy review process across GM Trusts for all participating Sites
- To reduce pharmacy set-up time across GM for all participating sites
- To improve GM pharmacy capacity to deliver more trials
- To improve Sponsors' satisfaction and attract more business into GM area
- To accelerate patient access to novel treatments

GM pharmacy review process synopsis

The GM clinical trials pharmacy risk assessment consists of 13 tabs, each focusing on a different aspect of the clinical trials pharmacy review.

The CRN will notify the GM pharmacy group of any new multicentre trials in the GM region.

A Trust/pharmacy unit should be nominated to lead on the risk assessment. This could be the first Trust which was selected as a site; however a later Trust may volunteer to lead on the RA, depending on capacity/availability to start work on the trial.

An email template is available for sending to sponsors to explain the collaborative working arrangement (Appendix 2)

The clinical trials pharmacist / senior technician from the leading pharmacy site or site with more capacity will complete the risk assessment from tab 1 to tab 12, following review of the trial specific documents and liaising with the sponsor/CRA regarding any pharmacy specific queries. Once this is completed, the risk assessment is then shared with any other participating sites in GM, who will complete the 'Site Specific Details' tab (tab 13) as per local requirements.

A dedicated tab for amendments has also been included in the risk assessment to allow processing of amendments using the single review approach used for study set-up.

Guidelines on how to complete the risk assessment

The risk assessment is composed of **13 sections (Tabs): 1. Initial review, 2. Study Details, 3. Amendments, 4. IMP details, 5. NIMP detail, 6. ATIMP, Early phase & GMO, 7. Injectable preparation 8. Excess treatment costs, 9. Pharmacy requirements 10. Homecare 11. Blinding-Unblinding, 12. Summary of Risks, 13. Site specific details.** Each section / tab of the risk assessment focuses on a different aspect of the pharmacy review to make navigation through the form easier for users. Detailed instructions on how to complete this form are explained below.

Roles and responsibilities

- It is the responsibility of the clinical trials pharmacist / senior technician who complete the assessment to ensure that the risk assessment is updated with the latest trial documents and amendments before sharing it to other participating sites
- It is the responsibility of the site completing the review to fill sections 1-12 of the risk assessment, although this can be done in collaboration with the other sites where appropriate
- It is the responsibility of the other participating site/s to ensure that they have the latest version of all trial documentation and risk assessment
- Each participating site is responsible for completing **tab 13 "Site specific"** as per individual site needs

General considerations

- Before starting the pharmacy review, ensure that all the documents used for the review are up to date.
- When completing the risk assessment, ensure that the information provided will be applicable to all participating sites (i.e. outsourced facilities such as an external aseptic unit)
- The risk assessment can be completed while waiting for regulatory approvals; however, it **can only be implemented** when approvals are received.
- **DO NOT delete tabs or rows from the risk assessment.**

- If a tab is not relevant, please indicate this at the top of each tab by using the specific drop down option.
- Drop-down options are provided when a standard answer is expected.
- The “date received” sections should be completed using the DD/MM/YYYY format.
- Risk assessments are version controlled. Each version should be numbered **in tab 1 - initial review** using the following format: *number.0*, starting with 1.0 and progressing in numerical order.
  Every Amendment (prior trial opening and when a trial is active) should be added in **Tab 3 - Amendments** and any impacting information should be updated across the risk assessment in the relevant sections.

Instructions on how to complete the risk assessment

A community of practice has been established across GM, where CT pharmacists and senior technicians meet to discuss new trials in feasibility/set up across the region, and which site would be leading on completing the pharmacy risk assessment.

The pharmacist / senior technician completing the risk assessment should collate all relevant documents from R&I or directly from Sponsor / CRA team.. A full list of required documents is provided in **appendix 1**.

Tab 1. Initial review

- This section includes information regarding the pharmacy professional completing the risk assessment, participating trusts across GM and UK, trials documents reviewed for completing the risk assessment and contact details of sponsor / CRO.
- **All** superseded versions should be documented in the *Superseded version* field in numerical order
- For ‘documents reviewed and approvals received’, ensure the field "Date received" is completed when sharing the risk assessment with other participating sites.
- If approvals have not been received at the time of sharing with new sites, complete section with ‘**Awaiting approvals’** and highlight section in red.

Tab 2. Study details

- This section includes general details of the study, including name / acronym, IRAS number, funding, sponsor name and study design.

Tab 3. Amendments

- Both substantial amendments and non-substantial amendments should be added in this section under the exact numbering format given by Authorities (e.g. NSA 1.0 or NSA 1) and in the order received – with the latest at the end.
- This section can be used for amendments that have been issued either before or after trial opening.

- The pharmacy professional reviewing the amendment should complete tab 3 and all the sections of the risk assessment that have been affected by the amendment. If any new risk and associated actions to be taken to minimise risks have been identified please include them in the dedicated lines at the bottom of each tab. These risks will automatically pull through from the risk section to **tab 12 - summary of risks.**
- **Approvals section**: Ensure Date received is completed when sharing with other sites.

Tab 4. IMP Details

- This section should be completed in line with information provided in the trial documentation. If information is missing, the pharmacy professional should liaise with CRA/Sponsor for clarification and keep a record of emails/ communication for audit purposes.
- If risks are identified while completing this section, these should be documented in the **Risk Identified** and **Actions to be taken to minimise risk** lines at the end of this section.
- When completing the **Actions to be taken to minimise risks** line, the pharmacy professional should provide a solution that can apply to all sites. If it is not possible to find a solution that can be implemented at all sites this, should be addressed at the local level. An appropriate comment should be included in this section, for example: "*To be reviewed by each site as per local requirement".*

Tab 5. NIMP Details

- See guidance for Tab 4

Tab 6. ATIMP, Early Phase and GMO

- This section is to be completed if the study is an early phase trial or includes the use of ATIMP(s) / GMO(s) and should be completed in conjunction with **Tab 4. IMP Details**
- If this section is not relevant to the trial, please indicate this at the top of the tab using the specific drop-down list.

Tab 7. Injectable preparation

- This section focuses on IMPs that require an injectable preparation, with the aim to establish method of preparation and administration as well as facilities and ancillaries required for preparation. In terms of ancillaries, it is important to establish whether hospital stocks can be used or will be sponsor provided.
- If this section is not relevant to the trial, please indicate this at the top of the tab using the specific drop-down list.

- If information is not available within the documentation provided, the pharmacy professional should liaise with Sponsor/CRO for clarifications.
- If local site arrangements are necessary to meet trial standards, then this should be documented in the relevant fields and recorded in the **"Risk identified"** section at the end of the tab. For example, if supply is subjective to each site (some sites might have different availabilities for supplying consumables depending on local processes) then this should be documented in the **"Risk identified" section.**
  An appropriate comment should be included in the "**Action to be taken to minimise risks"** section, for example: "*To be reviewed by each site as per local requirement".*

Tab 8. Excess treatment costs

- This section includes all additional costs that NHS sites may need to cover to deliver the trial. This includes, but it is not limited to, pharmacy out of hours working, use of high cost or rescue medicines, postage / courier costs and end of trial arrangements. It should be checked in conjunction with the NCVR spreadsheet.

  If local site arrangements are necessary, then this should be documented in the relevant field and recorded in the **"Risk identified"** section at the end of the tab. An appropriate comment should be included in the "**Action to be taken to minimise risks"** section, for example: "*To be reviewed by each site as per local requirement".*

Tab 9. Pharmacy requirements

- This section addresses all tasks directly managed by pharmacy for the delivery of the trial including randomisation, pharmacy clinical checks, dispensing, accountability, supply, return and disposal.
- This section should be completed in line with the information provided in the study documentation. If local arrangements are necessary for specific sites, this should be documented in the relevant section, identified as a risk and the **Action to be taken to minimise risks** section should be completed with "*To be reviewed by local site as per requirements'*

Tab10. Homecare

- This section lists information related to Homecare requirements.
- If this section is not relevant to the trial, please indicate this at the top of the tab using the specific drop-down list.

Tab 11. Blinding-Unblinding

- This section addresses blinding information and how unblinding will be managed.
- If local arrangements are necessary, these should be documented in the relevant section, identified as a risk and the **Action to be taken section to minimise risks** should be completed with *To be reviewed by local site as per requirements'*

Tab 12. Summary of risks

- This section summarises all risks identified whilst completing the risk assessment. Any information documented in the 'Risk(s) identified' and 'Action to be taken to minimise risk' fields at the end of each tab will be automatically populated in this tab.
- The pharmacy professional should identify solutions to mitigate identified risks (e.g.: contact CRA for clarification, provide outcome of discussion, etc). A record of steps taken should be kept for traceability and auditing purposes.
- **Each site is responsible for assessing and addressing risks documented in this section as well as make any site specific arrangements as per tab 13 - Site specific details.**

Tab 13. Site specific details

- This section should be completed by the Participating Site after receiving the risk assessment with tabs 1-12 completed by the leading pharmacy site.
- The contents and structure of this tab can be modified as needed, if a site decides to incorporate it in their existing trial set-up process. Please note, this is the ONLY tab that can be amended based on the needs of each site.
- To print the risk assessment in its entirety (i.e. for filing, if this is standard procedure at site), ctrl+select each tab and print. The print area is already pre-set for the document, so adjustments are not needed.

Related documents

Appendix 1

Initial documents request/pharmacy feasibility

Dear xxxx,
I am the clinical trial pharmacist working on the setup of the xxxx trial in pharmacy at the XXX. To facilitate our feasibility and risk assessment for the trial, could I please request the following documents and information:

Documents:

- Protocol
- Pharmacy manual / sample of prescription
- IB
- MSDS – for all IMPs in the study
- Patient facing documents, PIS/ICF/DIARIES
- Any accountability logs – Master/Subject specific/any other. Can we use our own?
- Sample labels
- Photograph of IMP if not in the protocol/pharmacy manual
- Any training slides
- Any extra forms, such as temperature excursion forms

Queries:

- What is the estimated number of patients for this trial at each GM site?
- Who are the PIs / CRAs?
- What's the recruitment period
- Is the IMP licensed in the UK? (any implications with NICE/availability to patients after trial)
- Can IRT email allocation be sent ahead of clinic/dispensing visit

Kind regards,

Appendix 2

Collaborative working arrangement email to sponsor

Dear Sponsor,

As part of a new CRN backed initiative to streamline the set-up process of Clinical Trials in Pharmacy, we will be working collaboratively with other Trusts across Greater Manchester to complete a number of key set-up tasks for this trial.

Only NHS Trusts in Greater Manchester which have a CDA in place for this trial and have received confirmation that they have been selected as a site, will take part in the collaborative working approach.

We hope that this new way of working will allow for faster and more efficient set-up of trials in Pharmacy.

Please do not hesitate to get in touch if you have any questions or concerns.

Kind Regards,
